# Association between periodontal disease and prostate disease: a mini review

**DOI:** 10.3389/fcimb.2025.1669490

**Published:** 2025-10-08

**Authors:** Shuxin Li, Hongliang Cao, Yueqiu Zhang, Tong Yang, Mingxuan Li, Chensen Lv, Xin Lian

**Affiliations:** ^1^ Department of Urology, The First Hospital of Jilin University, Changchun, China; ^2^ Department of Otorhinolaryngology, Head and Neck Surgery, West China Hospital, Sichuan University, Chengdu, China; ^3^ Department of Medicine, Yanbian University, Yanbian, China

**Keywords:** periodontal disease, oral microbiota, prostate cancer, benign prostatic hyperplasia, prostatitis, systemic inflammation, inflammatory factors

## Abstract

Periodontal disease (PD) is one of the most common chronic diseases in the oral cavity, typically referring to chronic inflammation caused by infection with pathogenic microorganisms in the oral cavity. PD primarily affects the tissues surrounding the teeth, leading to inflammation of the gums and periodontal tissues, destruction of the alveolar bone, and even loosening of the teeth. Numerous studies have shown that PD is not limited to the oral cavity but is also associated with the occurrence of diseases in multiple systems throughout the body. In recent years, increasing attention has been directed toward the interaction between PD and prostate diseases. This article reviews the potential associations between PD and prostate conditions such as chronic prostatitis, benign prostatic hyperplasia (BPH), and prostate cancer (PCa). It explores the pathological mechanisms underlying this interaction and its clinical implications. Additionally, this article aims to identify potential pathogenic mechanisms and propose possible approaches for preventing and treating prostate diseases through the management of PD.

## 1 Introduction

Prostate diseases are common urological conditions, primarily including non-cancerous prostate conditions such as prostatitis and benign prostatic hyperplasia (BPH), as well as prostate cancer (PCa). The global burden of prostate diseases is significant, particularly among middle-aged and older men (Gupta et al., 2017; Al-Ghazawi et al., 2023; Calderone et al., 2023). Prostate diseases impose a substantial economic burden on society and severely impact men’s quality of life and survival time (Calderone et al., 2023). Prostatitis can occur in men of all ages, with approximately 5–9% of adult men worldwide affected by prostatitis (Li et al., 2020; He et al., 2023). According to the classification system of the National Institutes of Health (NIH), prostatitis can be categorized into acute bacterial prostatitis, chronic bacterial prostatitis, chronic prostatitis, chronic pelvic pain syndrome (CP/CPPS), and asymptomatic prostatitis. Whether acute or chronic, patients with bacterial prostatitis may experience prominent lower urinary tract symptoms (LUTS) and urinary discomfort (Lam and Stokes, 2024). Patients with acute bacterial prostatitis may present with systemic infection symptoms, such as high fever, chills, severe pain in the perineum or suprapubic region, and a swollen, tender prostate palpable during digital rectal examination (Schoeb et al., 2017; Lam and Stokes, 2024; Yang et al., 2024). Chronic bacterial prostatitis, on the other hand, is characterized by recurrent urinary tract infection symptoms, including dull pain in the lower abdomen, and may be accompanied by sexual dysfunction (Schoeb et al., 2017; Lam and Stokes, 2024). The primary clinical symptom of chronic pelvic pain syndrome is persistent pelvic pain lasting ≥3 months, often radiating to the lower back, and frequently accompanied by urinary and sexual dysfunction (Hervás-Pérez et al., 2020; Matsukawa et al., 2022; Stevens et al., 2023). The incidence of BPH increases significantly with age, affecting over half of men over 50 years old (Yu et al., 2020; Calderone et al., 2023; Wang et al., 2025). BPH primarily manifests as non-cancerous enlargement of the prostate gland. The enlarged prostate gland compresses the urethra, causing bladder outlet obstruction and resulting in lower urinary tract symptoms such as weak urine flow, difficulty urinating, and a sensation of incomplete emptying (Sundaram et al., 2017; Zattoni et al., 2017; Dias et al., 2021; Liu et al., 2023; Kaltsas et al., 2025). In severe cases, it can lead to acute urinary retention and recurrent urinary tract infections (Kang et al., 2016; Zattoni et al., 2017; Kim EH. et al., 2018). PCa is the second most common malignant tumor in men globally, following lung cancer (Hussain et al., 2021). The incidence and mortality rates of prostate cancer have significantly increased in recent years, particularly among older men. Common clinical manifestations of PCa include increased nocturia, weakened urine flow, erectile dysfunction, and gross hematuria (Merriel et al., 2018). If PCa metastasizes to the spine and pelvis, it can cause metastatic bone pain (Yamashita et al., 2018). When it invades the spinal cord and nerves, it can lead to neurological symptoms (Viglialoro et al., 2021). However, due to the insidious nature of early symptoms, some cases are already at an advanced stage by the time of diagnosis, increasing treatment difficulty. Although prostate diseases have a high prevalence in the population and significantly impact men’s health, the factors influencing their occurrence and progression remain unclear.

Periodontal disease (PD) is a common, chronic inflammatory condition of the oral cavity, primarily comprising gingivitis or *periodontitis* (Zhao et al., 2024). PD often affects the supporting tissues surrounding the teeth, including the gums, periodontal ligaments, cementum, and alveolar bone. The development of PD is triggered by a complex inflammatory response resulting from an imbalance of oral microorganisms, which is associated with the immune response to pathogenic microorganisms and their toxins (Zhang et al., 2025). The human oral cavity harbors a diverse array of microorganisms, including bacteria, fungi, viruses, and protozoa, collectively referred to as the oral microbiota (Chattopadhyay et al., 2023). A normal microbiota maintains oral environmental stability, regulates oral pH levels, and stimulates the activation of immune-protective functions in the oral mucosa (Chattopadhyay et al., 2023). However, these microorganisms can also cause oral diseases; an increase in pathogenic bacteria and their metabolites may contribute to tooth destruction and oral tissue inflammation (Zhang et al., 2025). Additionally, oral microorganisms can enter the bloodstream and produce inflammatory factors, potentially triggering diseases in multiple systems throughout the body (Li et al., 2022). Recent studies have shown that PD caused by dysbiosis of the oral microbiota plays a significant role in the onset and progression of prostate diseases. In this review, we summarize epidemiological and pathophysiological studies on the relationship between PD and prostate diseases to explore potential methods for preventing and treating prostate diseases through PD intervention.

## 2 Observational and experimental evidence suggest a strong association between PD and prostate disease

Recent observational studies have shown that the oral health of male patients with prostate disease differs significantly from that of healthy males. This suggests that PD may play an essential role in the development, progression, and treatment outcomes of prostate disease. Additionally, several animal studies have reported similar findings (**Table 1**).

**Table 1:** Summary of evidence on the association between PD and prostate diseases, stratified by study design.

Research type	Prostate disease	First author/Year	Subject	Sample size	Research type	Main outcome
Observational						
Cohort Studies	Prostatitis	Fu et al., 2021	human	13,530	Cohort	Periodontitis patients had a higher risk of developing prostatitis.
	BPH	Kwon et al., 2025	human	79,497	Cohort	Periodontitis increased the risk of developing BPH within 1 year.
	PCa	Dizdar et al., 2017	human	280	Prospective Cohort	Men with periodontitis had a significantly increased risk of prostate cancer.
	Prostatitis	Lee et al., 2017	human	2,013	Longitudinal Cohort	Periodontal disease was associated with a 14% increased risk of prostatitis.
Case-Control	Prostatitis	Boyapati et al., 2018	human	100	Case-Control	Periodontal parameters were significantly elevated in patients with moderate to severe prostatitis.
	PCa	Vitor et al., 2025	human	372	Case-Control	The prevalence and severity of periodontitis were higher among prostate cancer cases.
Cross-Sectional	BPH	Wu et al., 2019	human	4,930	Cross-Sectional	PD increased the risk of BPH by 1.68 times.
Molecular	Prostatitis/BPH	Estemalik et al., 2017	human	24	16S rRNA sequencing	Oral pathogens (e.g., P. gingivalis, T. denticola) were detected in both prostate secretions and plaque samples.
	BPH/PCa	Alluri et al., 2021	human	90	16S rRNA sequencing	The expression of Corynebacterium was significantly higher in BPH, inflammation, and cancer tissues.
Mendelian randomization						
	BPH	Wei et al., 2024	human	284,358	MR	No evidence of a causal relationship between periodontitis and BPH was observed.
	BPH/Prostatitis	Yang et al., 2025	human	489,422	MR	No causal relationship between periodontitis and prostate disease was found.
Experimental						
Animal Studies	BPH	Wang et al., 2024	human/rat	–	Animal experiments	P. gingivalis infection upregulated inflammatory cytokines and activated the Akt pathway, associated with BPH.
	PCa	Wang et al., 2024	rat	–	Animal experiments	Periodontitis promoted tumor growth and immune escape via PD-1/PD-L1, attenuated by anti-PD-1 therapy.
Cell Studies	PCa	Groeger et al., 2022	PCa cells	–	Cell experiments	P. gingivalis infection induced PD-L1 upregulation via NOD1/NOD2, promoting immune evasion.
Meta-analysis	PCa	Guo et al., 2021	human	440,911	Meta-analysis	Periodontal disease significantly increased the risk of prostate cancer by 1.40 times. No significant increase was found in treated patients.

### 2.1 PD and prostatitis

PD is a chronic inflammatory disease, and its inflammatory mediators may affect distant organs through the bloodstream. For example, PD may influence the development of prostatitis. In a clinical study, 100 patients with both PD and chronic prostatitis were grouped, and periodontal parameters and serum PSA levels were recorded separately (Boyapati et al., 2018). It was found that periodontal parameters were significantly elevated in patients with moderate to severe prostatitis, indicating a pathological association between periodontitis and chronic prostatitis. In a cohort study in Taiwan comparing individuals with chronic periodontitis and healthy individuals, the risk of prostatitis was significantly higher in periodontitis patients than in those without periodontitis (Fu et al., 2021). Furthermore, John Estemalik et al. also identified similar *DNA from Porphyromonas gingivalis and Treponema denticola* in prostate secretions and subgingival plaque from the same individuals with chronic prostatitis (Estemalik et al., 2017). These observational studies suggest that oral pathogens could potentially influence prostatitis through hematogenous spread or direct diffusion, confirming the association between PD and prostatitis.

### 2.2 PD and BPH

BPH is the most common urinary tract disease in elderly men, mainly manifested as cell proliferation in the prostate tissue, leading to an enlarged prostate. There may be a potential link between PD and the occurrence of BPH. A cross-sectional study of 4,930 men found that PD increased the risk of BPH by 1.68 times, with periodontitis patients having an even higher risk (Wu et al., 2019). In this study, the population was also grouped based on prostate volume, and PD was still found to increase the risk of BPH in different subgroups. However, individuals with a prostate volume >60g had a higher risk of PD (Wu et al., 2019). A cohort study in South Korea found that participants with a history of recurrent periodontitis had a higher incidence of BPH, indicating that chronic periodontitis is a risk factor for BPH (Kwon et al., 2025). Additionally, another study found that patients who underwent periodontal treatment had a reduced risk of BPH (Fu et al., 2021). In patients with both BPH and periodontitis, the same oral pathogens were detected in both prostate fluid and subgingival plaque (Estemalik et al., 2017). These studies suggest that PD may contribute to the development of BPH in men. However, other studies have yielded differing results, and Mendelian randomization studies on PD and BPH have not confirmed causal relationships at the genetic level (Wei et al., 2024; Yang L. et al., 2025). Future prospective cohort studies with larger sample sizes may be needed to clarify the true association between PD and BPH.

### 2.3 PD and PCa

Prostate cancer (PCa) is one of the most common malignant tumors in men. The association between PD and PCa has been confirmed in numerous studies. In a case-control study involving 152 PCa patients and 220 control cases, periodontal examinations revealed a higher prevalence of PD among PCa patients, suggesting a link between periodontitis and the development of PCa (Vitor et al., 2025). In a 12-year longitudinal cohort study in South Korea, PD was found to increase the risk of PCa by 14%, with a significant positive correlation between PD and PCa (Lee et al., 2017). Additionally, Omer Dizdar et al. conducted a 12-year follow-up study on 280 participants with moderate to severe periodontitis, finding that periodontitis patients had a higher risk of developing PCa and blood cancer (Dizdar et al., 2017). A meta-analysis of cohort studies on the association between PD and PCa found that PD is associated with an increased risk of PCa. However, no significant association was observed in patients who received periodontal treatment (Guo et al., 2021). In another study, using 16S rRNA as primers, genomic DNA analysis of PCa glandular tissue identified specific periodontal pathogens, such as *Fusobacterium nucleatum* (Alluri et al., 2021). These study results support the view that PD is a risk factor for PCa. Middle-aged and older men should enhance their awareness of oral health and implement preventive and therapeutic measures for PD to reduce the risk of PCa.

## 3 Potential biological mechanisms linking PD and prostate disease

**Figure 1:**
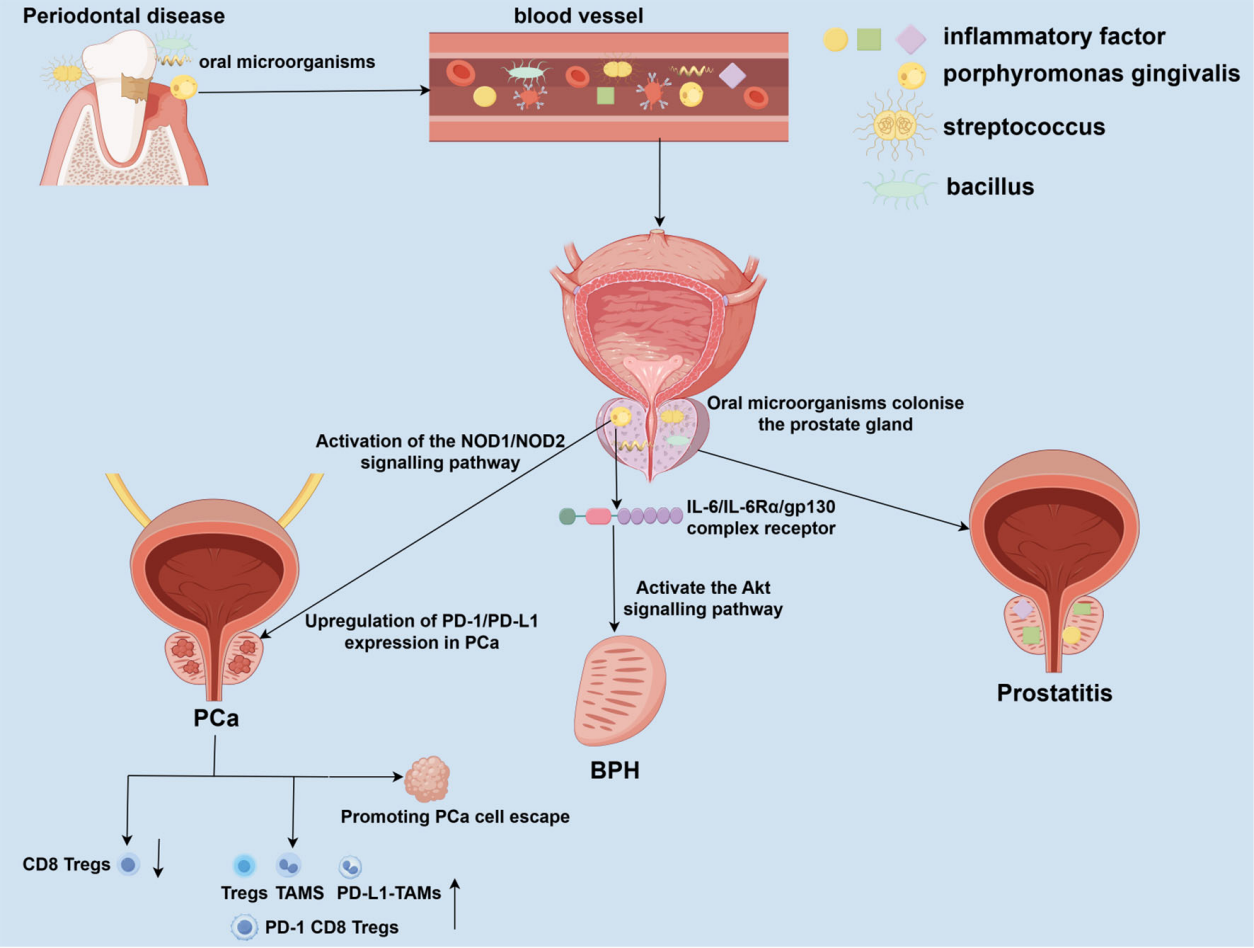
Potential mechanisms linking periodontal disease to prostate disease pathogenesis. Periodontal disease (PD), driven by oral dysbiosis, may impact prostate health through direct and indirect pathways. Oral pathogens from compromised periodontal pockets can enter the circulation and disseminate to the prostate gland. Colonization and infection by these pathogens can directly incite local inflammation, contributing to prostatitis. PD serves as a reservoir of chronic inflammation, releasing pro-inflammatory mediators and components of periodontal pathogens, such as lipopolysaccharide (LPS), into the bloodstream. This systemic inflammatory milieu can promote a pro-inflammatory microenvironment within the prostate. Infection with P. gingivalis and its LPS upregulates inflammatory cytokines, activates the IL-6/IL-6Rα/gp130 complex, and subsequently stimulates the Akt signaling pathway. This cascade disrupts the balance between cell proliferation and apoptosis, favoring prostate tissue hyperplasia. P. gingivalis infection, mediated through pattern recognition receptors, can upregulate immune checkpoint molecules like PD-L1 on tumor cells. This, along with the systemic inflammatory response, contributes to an immunosuppressive TME characterized by increased TAMs, regulatory Tregs, and exhausted CD8+ T cells, ultimately facilitating tumor immune evasion and progression. PD, periodontal disease; BPH, benign prostatic hyperplasia; PCa, prostate cancer; IL-6, interleukin-6; IL-6Rα, interleukin-6 receptor alpha; gp130, glycoprotein 130; LPS, lipopolysaccharide; Akt, protein kinase B; NOD1/2, nucleotide-binding oligomerization domain-containing protein 1/2; PD-L1, programmed cell death ligand 1; TME, tumor microenvironment; TAMs, tumor-associated macrophages; Tregs, regulatory T cells.

Although current observational studies have identified several associations between PD and prostate disease, and the differences described in these studies may become targets for the prevention and treatment of prostate disease, their specific mechanisms of action remain to be further clarified. This section will discuss in detail the potential mechanisms of action between PD and prostate disease, as well as how PD affects the development of prostate disease (**Figure 1**). It has been hypothesized that PD may directly or indirectly affect the onset and progression of prostate disease through the oral-prostate axis (Estemalik et al., 2017; Alluri et al., 2021; Wang SY. et al., 2024). One proposed mechanism suggests that oral pathogens may enter compromised periodontal tissues and subsequently enter and colonize the prostate via the blood or lymphatic systems (Estemalik et al., 2017; Alluri et al., 2021; Wang SY. et al., 2024). Moreover, PD is a chronic inflammatory disease, and the inflammatory factors it releases may affect prostate tissues through blood circulation, leading to a chronic inflammatory microenvironment, which in turn promotes the transformation of prostate disease from inflammation to hyperplasia or cancer. In recent years, with the advancement of biotechnology, researchers have gradually discovered the phenomenon of oral microbial transfer. In one study, 30 prostate tissue samples were selected from patients with inflammation, benign hyperplasia, and cancer (Alluri et al., 2021). Using 16S rRNA as primers, DNA from periodontal pathogens was detected in all these tissues (Alluri et al., 2021). Additionally, Wang et al. cultured subgingival plaque and prostate fluid from patients with BPH complicated by periodontitis, performed 16S rDNA sequencing, and detected oral pathogens such as *Porphyromonas gingivalis*, *Streptococcus oralis*, and *Fusobacterium nucleatum (* Wang SY. et al., 2024). In animal experiments, researchers established rat models of periodontitis and benign prostatic hyperplasia (BPH), as well as their co-morbidities, to explore their interrelationships. Analysis of the gut microbiota by 16S rDNA sequencing and LC-MS/MS-based fecal metabolomics demonstrated that periodontitis may contribute to the pathogenesis of BPH by inducing dysbiosis of the gut flora and altering microbial-derived metabolites (Guo et al., 2023). When these periodontal pathogens colonize and infect the prostate, they may cause local inflammation and induce the onset of prostatitis. Infection of the prostate with *Porphyromonas gingivalis* upregulates the expression of inflammatory cytokines interleukin-6 (IL-6), interleukin-6 receptor-α (IL-6Rα), and glycoprotein 130 (gp130) in the tissue. Its toxic factor, *Porphyromonas gingivalis* lipopolysaccharide (P.g-LPS), activates the Akt pathway through the IL-6/IL-6Rα/gp130 complex, significantly inhibiting prostate cell apoptosis and proliferation while promoting cell mitosis and proliferation, leading to BPH (Wang SY. et al., 2024). Additionally, *Porphyromonas gingivalis* and its LPS can upregulate PD-1/PD-L1 expression in PCa cells via the NOD1/NOD2 signaling pathway, promoting the increase in the proportion of tumor-associated macrophages (TAMs), regulatory T cells (Tregs), PD-L1 TAMs, and PD-1 CD8 Tregs, while reducing the proportion of CD8 Tregs (Groeger et al., 2022; Wang S. et al., 2024). This alters the tumor microenvironment to promote tumor immune escape and may contribute to the development and progression of PCa (Groeger et al., 2022; Wang S. et al., 2024).

## 4 Discussion

This review focuses on studies investigating the association between PD and prostate disease, as well as the potential mechanisms by which periodontal disease and its common oral pathogens influence the onset and progression of prostate disease (**Figure 1**). We propose that controlling PD may reduce the risk of developing prostate disease. This review primarily covers epidemiological studies on the association between PD and prostate disease, as well as a limited number of *in vivo* and *in vitro* studies. Epidemiological studies mainly include case-control studies and cross-sectional studies. *In vivo* studies primarily use rat or mouse animal models to simulate how PD-associated pathogens and inflammatory factors promote the development of prostate diseases. *In vitro* studies mainly involve microbiome and metabolome research, utilizing 16S rRNA sequencing of prostate-related tissues and subgingival exudate (Estemalik et al., 2017).

When interpreting the epidemiological association between PD and prostate diseases, it is essential to consider the influence of multiple potential confounding factors carefully. These confounding variables may partially drive the correlations observed in observational studies. Age represents the most clearly identified common risk factor shared by prostate diseases and PD. Advanced age is not only associated with prostate tissue hyperplasia and the accumulation of genetic mutations. Still, it is also closely linked to the physiological degeneration of periodontal tissues and prolonged microbial exposure (Tan et al., 2021; Fox et al., 2023; Bertolini and Clark, 2024). Furthermore, smoking is a well-established risk factor for periodontitis while also inducing systemic inflammation and oxidative stress (Mazurek-Mochol et al., 2017; Ehsan, 2025). Research indicates smoking may independently increase prostate cancer risk or influence its progression. Smokers typically exhibit poorer oral health and may face distinct prostate disease risks compared to non-smokers, making smoking a significant potential confounder (Tang et al., 2017; AlQobaly et al., 2022; Huang et al., 2024; Yang X. et al., 2025). Diabetes is also strongly associated with more severe and extensive periodontitis, exacerbating periodontal destruction through mechanisms such as advanced glycation end-product (AGEs) accumulation and immune dysfunction (Liu et al., 2018; Nonaka et al., 2018). Simultaneously, diabetes is recognized as a risk factor for BPH (Ozcan et al., 2017). The chronic inflammatory state accompanying diabetes may represent an upstream pathway linking PD to prostate diseases (Chen and Feng, 2023). Socioeconomic status (SES) is a multidimensional factor encompassing education, income, occupation, and access to healthcare. Lower SES is typically associated with poorer oral hygiene, reduced dental care utilization, higher smoking rates, unhealthy diets, and inadequate management of systemic diseases (Fowke et al., 2008; Kim HN. et al., 2018; Park et al., 2018; Tadakamadla et al., 2020; Mikami et al., 2023). These factors may contribute to the elevated burden of both PD and prostate disease in this population, introducing confounding into analyses (Fowke et al., 2008; Kim HN. et al., 2018; Park et al., 2018; Tadakamadla et al., 2020; Mikami et al., 2023). Although most observational studies attempt to control these variables through multivariate regression models, residual confounding may persist. Furthermore, Mendelian randomization studies have failed to establish a genetic-level causal relationship between PD and BPH, which partially weakens the causal inferences drawn from observational findings. This suggests that such associations may be influenced by the confounding factors or reverse causality.

Recent studies have shown that PD is closely associated with prostate diseases, including chronic prostatitis, BPH, and PCa. Research indicates that periodontal disease may affect prostate health through microbial transmission and systemic inflammation, highlighting the importance of oral health in preventing the risk of prostate diseases. Oral pathogens, such as *Porphyromonas gingivalis* and *Fusobacterium nucleatum*, have been detected in prostate tissue, supporting the hypothesis that these pathogens may spread from the oral cavity to the prostate via the bloodstream or lymphatic system (Alluri et al., 2021). These pathogens may not only directly induce prostate inflammation but also promote cancer development by altering the local microenvironment. For example, *Porphyromonas gingivalis* has been shown to upregulate the expression of inflammatory cytokines and activate the Akt signaling pathway and the PD-1/PD-L1 receptor pathway, which are closely associated with the development of BPH and PCa (Groeger et al., 2022; Wang SY. et al., 2024). Furthermore, chronic inflammation caused by periodontal disease can exacerbate prostate lesions by releasing pro-inflammatory mediators such as IL-6 and reactive oxygen species into the bloodstream (Tonguç et al., 2022; Wang S. et al., 2024). This systemic response may sustain a pro-inflammatory environment within the prostate, thereby inducing tissue hyperplasia or malignant transformation (Groeger et al., 2022; Wang S. et al., 2024). The observed reduction in the risk of benign prostatic hyperplasia following periodontal treatment further underscores the critical role of inflammation in disease progression (Fu et al., 2021). Although most observational studies support a positive correlation between PD and prostate disease, considerable heterogeneity exists between studies, with inconsistent and even contradictory conclusions emerging, particularly in BPH research. For instance, cross-sectional and cohort studies by Wu et al. and Kwon et al. both indicated that PD significantly increases the risk of BPH, whereas studies by Wei et al. and Yang et al., based on Mendelian randomization (MR) analysis, failed to identify a genetic causal relationship between the two (Wu et al., 2019; Wei et al., 2024; Kwon et al., 2025; Yang L. et al., 2025). These inconsistencies may stem from several factors. Observational studies are susceptible to confounding variables, while MR analysis, although it reduces confounding, remains sensitive to weak instrumental variables or differences in population structure. Furthermore, inconsistencies in diagnostic criteria for BPH and severity classification of PD across studies may introduce bias. Some studies were based on Asian populations, while others utilized European databases; differences in genetic backgrounds, environmental factors, and healthcare accessibility may affect the generalizability of the findings.

This study has several limitations, primarily focused on epidemiological and *in vitro* experimental aspects, with limited involvement *in vivo* experiments. The specific molecular pathways by which bacterial translocation and oral pathogens induce prostatitis and carcinogenesis require further elucidation using advanced animal models and multi-omics approaches. Additionally, while the potential of PD management to alleviate the burden of prostate disease appears promising, it has not been fully explored. However, there is currently a complete lack of evidence from randomized controlled trials (RCTs) to substantiate that treating periodontal disease can alleviate symptoms of prostate conditions or alter their natural course. For instance, whether routine periodontal care for high-risk populations, such as elderly men, can reduce the incidence of PCa remains to be determined. Future research should utilize animal models that simulate the coexistence of PD and prostate disease to elucidate the molecular mechanisms and validate targeted therapeutic approaches. PD can facilitate the entry of oral pathogens into the bloodstream, causing transient bacteremia (Gualtero et al., 2023). While the existence of a resident blood microbiome in healthy individuals is debated and not supported by large-scale studies, it is plausible that during periods of active periodontal infection, these circulating pathogens could disseminate to and seed the prostate gland (Tan et al., 2023). Integrating multi-omics technologies, such as metagenomics and metabolomics, enables a systematic analysis of microbial characteristics and metabolite associations between oral and prostate dysbiosis. Furthermore, metagenomic sequencing of matched oral, blood, and prostate samples allows tracing the migration and colonization pathways of oral microbiota (Tan et al., 2023). Conducting additional randomized controlled trials to evaluate the impact of periodontal treatment on the severity of prostate symptoms or disease progression will provide stronger scientific evidence for clinical application.

## 5 Conclusion

The interplay between PD and prostate diseases highlights a potential shared pathophysiology centered on infection and inflammation. While current observational evidence suggests an association, it does not establish a causal relationship. Periodontitis may represent a novel, modifiable risk factor for prostate diseases. However, translating this association into clinical practice requires rigorous mechanistic studies to elucidate the causal pathways and, crucially, interventional trials to confirm that periodontal treatment can effectively improve prostate health outcomes. Enhancing oral health awareness and integrating dental care into urological health strategies may offer a promising avenue for the prevention and adjunctive therapy of prostate diseases, pending validation from future research.

## References

[B1] Al-Ghazawi M. Salameh H. Amo-Afful S. Khasawneh S. Ghanem R. (2023). An in-depth look into the epidemiological and etiological aspects of prostate cancer: A literature review. Cureus. 15, e48252. doi: 10.7759/cureus.48252, PMID: 38054148 PMC10694784

[B2] Alluri L. S. C. Paes Batista da Silva A. Verma S. Fu P. Shen D. L. MacLennan G.. (2021). Presence of specific periodontal pathogens in prostate gland diagnosed with chronic inflammation and adenocarcinoma. Cureus. 13, e17742. doi: 10.7759/cureus.17742, PMID: 34659955 PMC8492166

[B3] AlQobaly L. Abed H. Alsahafi Y. Sabbah W. Hakeem F. F. (2022). Does smoking explain the association between use of e-cigarettes and self-reported periodontal disease? J. Dent. 122, 104164. doi: 10.1016/j.jdent.2022.104164, PMID: 35580834

[B4] Bertolini M. Clark D. (2024). Periodontal disease as a model to study chronic inflammation in aging. Geroscience. 46, 3695–3709. doi: 10.1007/s11357-023-00835-0, PMID: 37285008 PMC11226587

[B5] Boyapati R. Swarna C. Devulapalli N. Sanivarapu S. Katuri K. K. Kolaparthy L. (2018). Unveiling the link between prostatitis and periodontitis. Contemp Clin. Dent. 9, 524–529. doi: 10.4103/ccd.ccd_746_18, PMID: 31772457 PMC6868634

[B6] Calderone C. E. Turner E. M. Hayek O. E. Summerlin D. West J. T. Rais-Bahrami S.. (2023). Contemporary review of multimodality imaging of the prostate gland. Diagnostics (Basel) 13, 1860. doi: 10.3390/diagnostics13111860, PMID: 37296712 PMC10252565

[B7] Chattopadhyay I. Lu W. Manikam R. Malarvili M. B. Ambati R. R. Gundamaraju R. (2023). Can metagenomics unravel the impact of oral bacteriome in human diseases? Biotechnol. Genet. Eng. Rev. 39, 85–117. doi: 10.1080/02648725.2022.2102877, PMID: 35861776

[B8] Chen G. Feng L. (2023). Analysis of platelet and monocyte-to-lymphocyte ratio and diabetes mellitus with benign prostatic enlargement. Front. Immunol. 14, 1166265. doi: 10.3389/fimmu.2023.1166265, PMID: 37492582 PMC10363740

[B9] Dias U. S. Jr. de Moura M. R. L. Viana P. C. C. de Assis A. M. Marcelino A. S. Z. Moreira A. M.. (2021). Prostatic artery embolization: indications, preparation, techniques, imaging evaluation, reporting, and complications. Radiographics. 41, 1509–1530. doi: 10.1148/rg.2021200144, PMID: 34415807 PMC9394104

[B10] Dizdar O. Hayran M. Guven D. C. Yılmaz T. B. Taheri S. Akman A. C.. (2017). Increased cancer risk in patients with periodontitis. Curr. Med. Res. Opin. 33, 2195–2200. doi: 10.1080/03007995.2017.1354829, PMID: 28699803

[B11] Ehsan H. (2025). The influence of smoking on periodontal health: A case-control study in Afghanistan. J. Periodontol. 96, 817–825. doi: 10.1002/JPER.24-0693, PMID: 39964200

[B12] Estemalik J. Demko C. Bissada N. F. Joshi N. Bodner D. Shankar E.. (2017). Simultaneous detection of oral pathogens in subgingival plaque and prostatic fluid of men with periodontal and prostatic diseases. J. Periodontol. 88, 823–829. doi: 10.1902/jop.2017.160477, PMID: 28548883

[B13] Fowke J. H. Murff H. J. Signorello L. B. Lund L. Blot W. J. (2008). Race and socioeconomic status are independently associated with benign prostatic hyperplasia. J. Urol. 180, 2091–2096. doi: 10.1016/j.juro.2008.07.059, PMID: 18804231 PMC2692430

[B14] Fox J. J. Hashimoto T. Navarro H. I. Garcia A. J. Shou B. L. Goldstein A. S. (2023). Highly multiplexed immune profiling throughout adulthood reveals kinetics of lymphocyte infiltration in the aging mouse prostate. Aging (Albany NY). 15, 3356–3380. doi: 10.18632/aging.204708, PMID: 37179121 PMC10449296

[B15] Fu E. Cheng C. M. Chung C. H. Lee W. C. Chen W. L. Sun G. H.. (2021). Association of chronic periodontitis with prostatic hyperplasia and prostatitis: A population-based cohort study in Taiwan. J. Periodontol. 92, 72–86. doi: 10.1002/JPER.19-0706, PMID: 32627845

[B16] Groeger S. Wu F. Wagenlehner F. Dansranjav T. Ruf S. Denter F.. (2022). PD-L1 up-regulation in prostate cancer cells by porphyromonas gingivalis. Front. Cell Infect. Microbiol. 12, 935806. doi: 10.3389/fcimb.2022.935806, PMID: 35846769 PMC9277116

[B17] Gualtero D. F. Lafaurie G. I. Buitrago D. M. Castillo Y. Vargas-Sanchez P. K. Castillo D. M. (2023). Oral microbiome mediated inflammation, a potential inductor of vascular diseases: a comprehensive review. Front. Cardiovasc. Med. 10, 1250263. doi: 10.3389/fcvm.2023.1250263, PMID: 37711554 PMC10498784

[B18] Guo Z. Gu C. Li S. Gan S. Li Y. Xiang S.. (2021). Periodontal disease and the risk of prostate cancer: a meta-analysis of cohort studies. Int. Braz. J. Urol. 47, 1120–1130. doi: 10.1590/s1677-5538.ibju.2020.0333, PMID: 33650836 PMC8486441

[B19] Guo X. P. Yang J. Wu L. Fang C. Gu J. M. Li F.. (2023). Periodontitis relates to benign prostatic hyperplasia via the gut microbiota and fecal metabolome. Front. Microbiol. 14, 1280628. doi: 10.3389/fmicb.2023.1280628, PMID: 38163068 PMC10756679

[B20] Gupta S. Gupta A. Saini A. K. Majumder K. Sinha K. Chahal A. (2017). Prostate cancer: how young is too young? Curr. Urol 9, 212–215. doi: 10.1159/000447143, PMID: 28413383 PMC5385860

[B21] He H. Luo H. Xu H. Qian B. Zou X. Zhang G.. (2023). Preclinical models and evaluation criteria of prostatitis. Front. Immunol. 14, 1183895. doi: 10.3389/fimmu.2023.1183895, PMID: 37228599 PMC10203503

[B22] Hervás-Pérez J. P. Jiménez Díaz-Benito V. Guodemar-Pérez J. Ruiz-López M. García-Fernández P. Rodríguez-López E. S.. (2020). The influence of physical activity as an alternative treatment to chronic prostatitis: A meta-analysis. Rev. Int. Androl. 18, 107–116. doi: 10.1016/j.androl.2018.12.001, PMID: 30871896

[B23] Huang Y. Q. Xu J. N. Huang Y. Xu Y. D. Wang H. L. Shi W. T.. (2024). Independent and combined effects of smoking, drinking and depression on periodontal disease. BMC Oral. Health 24, 535. doi: 10.1186/s12903-024-04287-6, PMID: 38711116 PMC11075253

[B24] Hussain Y. Mirzaei S. Ashrafizadeh M. Zarrabi A. Hushmandi K. Khan H.. (2021). Quercetin and its nano-scale delivery systems in prostate cancer therapy: paving the way for cancer elimination and reversing chemoresistance. Cancers (Basel) 13, 1602. doi: 10.3390/cancers13071602, PMID: 33807174 PMC8036441

[B25] Kaltsas A. Giannakodimos I. Symeonidis E. N. Deligiannis D. Stavropoulos M. Symeonidis A.. (2025). To rezūm or not to rezūm: A narrative review of water vapor thermal therapy for benign prostatic hyperplasia. J. Clin. Med. 14, 4254. doi: 10.3390/jcm14124254, PMID: 40565999 PMC12194174

[B26] Kang D. H. Cho K. S. Ham W. S. Choi Y. D. Lee J. Y. (2016). A systematic review and meta-analysis of functional outcomes and complications following the photoselective vaporization of the prostate and monopolar transurethral resection of the prostate. World J. Mens Health 34, 110–122. doi: 10.5534/wjmh.2016.34.2.110, PMID: 27574594 PMC4999484

[B27] Kim E. H. Brockman J. A. Andriole G. L. (2018). The use of 5-alpha reductase inhibitors in the treatment of benign prostatic hyperplasia. Asian J. Urol. 5, 28–32. doi: 10.1016/j.ajur.2017.11.005, PMID: 29379733 PMC5780290

[B28] Kim H. N. Jang Y. E. Kim C. B. Kim N. H. (2018). Socioeconomic status and self-reported periodontal symptoms in community-dwelling individuals: data from the Korea Community Health Surveys of 2011 and 2013. Int. Dent. J. 68, 411–419. doi: 10.1111/idj.12407, PMID: 29869334 PMC9379015

[B29] Kwon M. J. Kang H. S. Choi H. G. Kim J. H. Yoo D. M. Lee N. E.. (2025). Chronic periodontitis as a risk factor for benign prostatic hyperplasia: A cohort study. J. Clin. Med. 14, 1279. doi: 10.3390/jcm14041279, PMID: 40004810 PMC11857014

[B30] Lam J. C. Stokes W. (2024). Acute and chronic prostatitis. Am. Fam Physician. 110, 45–51. 39028781

[B31] Lee J. H. Kweon H. H. Choi J. K. Kim Y. T. Choi S. H. (2017). Association between Periodontal disease and Prostate cancer: Results of a 12-year Longitudinal Cohort Study in South Korea. J. Cancer. 8, 2959–2965. doi: 10.7150/jca.20532, PMID: 28928887 PMC5604447

[B32] Li G. Chang D. Chen D. Zhang P. You Y. Huang X.. (2020). Efficacy of radial extracorporeal shock wave therapy for chronic prostatitis/chronic pelvic pain syndrome: A protocol for systematic review. Med. (Baltimore). 99, e22981. doi: 10.1097/MD.0000000000022981, PMID: 33126371 PMC7598797

[B33] Li Y. Zhu M. Liu Y. Luo B. Cui J. Huang L.. (2022). The oral microbiota and cardiometabolic health: A comprehensive review and emerging insights. Front. Immunol. 13, 1010368. doi: 10.3389/fimmu.2022.1010368, PMID: 36466857 PMC9716288

[B34] Liu Y. Yu Y. Nickel J. C. Iwasaki L. R. Duan P. Simmer-Beck M.. (2018). Gender differences in the association of periodontitis and type 2 diabetes. Int. Dent. J. 68, 433–440. doi: 10.1111/idj.12399, PMID: 29786140 PMC9379021

[B35] Liu J. Zhang J. Fu X. Yang S. Li Y. Liu J.. (2023). The emerging role of cell adhesion molecules on benign prostatic hyperplasia. Int. J. Mol. Sci. 24, 2870. doi: 10.3390/ijms24032870, PMID: 36769190 PMC9917596

[B36] Matsukawa Y. Funahashi Y. Ishida S. Naito Y. Yuba T. Matsuo K.. (2022). Clinical features and urodynamic findings in elderly men with chronic prostatitis. Int. J. Urol. 29, 441–445. doi: 10.1111/iju.14805, PMID: 35146792

[B37] Mazurek-Mochol M. Majorczyk E. Banach J. Dembowska E. Kuśnierczyk P. Safranow K.. (2017). The influence of KIR gene presence/absence polymorphisms on the development of periodontal disease in smokers and non-smokers. Cent Eur. J. Immunol. 42, 347–353. doi: 10.5114/ceji.2017.72796, PMID: 29472811 PMC5820974

[B38] Merriel S. W. D. Funston G. Hamilton W. (2018). Prostate cancer in primary care. Adv. Ther. 35, 1285–1294. doi: 10.1007/s12325-018-0766-1, PMID: 30097885 PMC6133140

[B39] Mikami R. Mizutani K. Aoyama N. Matsuura T. Suda T. Takeda K.. (2023). Income-related inequalities in the association of smoking with periodontitis: a cross-sectional analysis in Tokyo Metropolitan Districts. Clin. Oral. Investig. 27, 519–528. doi: 10.1007/s00784-022-04747-9, PMID: 36241924

[B40] Nonaka K. Kajiura Y. Bando M. Sakamoto E. Inagaki Y. Lew J. H.. (2018). Advanced glycation end-products increase IL-6 and ICAM-1 expression via RAGE, MAPK and NF-κB pathways in human gingival fibroblasts. J. Periodontal Res. 53, 334–344. doi: 10.1111/jre.12518, PMID: 29193068

[B41] Ozcan L. Besiroglu H. Dursun M. Polat E. C. Otunctemur A. Ozbek E. (2017). Comparison of the clinical parameters of benign prostate hyperplasia in diabetic and non diabetic patients. Arch. Ital Urol Androl. 89, 26–30. doi: 10.4081/aiua.2017.1.26, PMID: 28403591

[B42] Park M. B. Hyun D. S. Song J. M. Chung H. C. Kwon S. W. Kim S. C.. (2018). Association between the symptoms of benign prostatic hyperplasia and social disparities: Does social capital promote prostate health? Andrologia 50, e13125. doi: 10.1111/and.13125, PMID: 30132961

[B43] Schoeb D. S. Schlager D. Boeker M. Wetterauer U. Schoenthaler M. Herrmann T. R. W.. (2017). Surgical therapy of prostatitis: a systematic review. World J. Urol. 35, 1659–1668. doi: 10.1007/s00345-017-2054-0, PMID: 28612108

[B44] Stevens R. H. Zhang H. Kajsik M. Płoski R. Rydzanicz M. Sabaka P.. (2023). Successful use of a phage endolysin for treatment of chronic pelvic pain syndrome/chronic bacterial prostatitis. Front. Med. (Lausanne). 10, 1238147. doi: 10.3389/fmed.2023.1238147, PMID: 37649979 PMC10462781

[B45] Sundaram D. Sankaran P. K. Raghunath G. Vijayalakshmi S. Vijayakumar J. Yuvaraj M. F.. (2017). Correlation of prostate gland size and uroflowmetry in patients with lower urinary tract symptoms. J. Clin. Diagn. Res. 11, Ac01–Aac4. doi: 10.7860/JCDR/2017/26651.9835, PMID: 28658743 PMC5483645

[B46] Tadakamadla S. K. Tadakamadla J. Kroon J. Lalloo R. Johnson N. W. (2020). Effect of family characteristics on periodontal diseases in children and adolescents-A systematic review. Int. J. Dent. Hyg. 18, 3–16. doi: 10.1111/idh.12398, PMID: 30941877

[B47] Tan J. Dai A. Pan L. Zhang L. Wang Z. Ke T.. (2021). Inflamm-aging-related cytokines of IL-17 and IFN-γ Accelerate osteoclastogenesis and periodontal destruction. J. Immunol. Res. 2021, 9919024. doi: 10.1155/2021/9919024, PMID: 34395635 PMC8357511

[B48] Tan C. C. S. Ko K. K. K. Chen H. Liu J. Loh M. Chia M.. (2023). No evidence for a common blood microbiome based on a population study of 9,770 healthy humans. Nat. Microbiol. 8, 973–985. doi: 10.1038/s41564-023-01350-w, PMID: 36997797 PMC10159858

[B49] Tang B. Han C. T. Gan H. L. Zhang G. M. Zhang C. Z. Yang W. Y.. (2017). Smoking increased the risk of prostate cancer with grade group ≥ 4 and intraductal carcinoma in a prospective biopsy cohort. Prostate. 77, 984–989. doi: 10.1002/pros.23354, PMID: 28422303

[B50] Tonguç M. Öztürk C. Polat G. Bobuşoğlu O. Tek S. A. Taşdelen B.. (2022). Investigation of the relationship between periodontal and systemic inflammation in children with Sickle Cell Disease: A case- control study. Cytokine. 149, 155724. doi: 10.1016/j.cyto.2021.155724, PMID: 34653827

[B51] Viglialoro R. Esposito E. Zanca R. Gessi M. Depalo T. Aghakhanyan G.. (2021). What to trust, PSA or [(68)Ga]Ga-PSMA-11: learn from experience. Res. Rep. Urol. 13, 597–601. doi: 10.2147/RRU.S316446, PMID: 34447724 PMC8384575

[B52] Vitor G. P. Carvalho A. P. Esteves Lima R. P. Miconi W. G. Costa F. O. Cota L. O. M. (2025). Association between periodontitis and prostate cancer: A case-control study. J. Periodontol. 96:1026–1034. doi: 10.1002/JPER.24-0440, PMID: 40254848

[B53] Wang S. Y. Cai Y. Hu X. Li F. Qian X. H. Xia L. Y.. (2024). P. gingivalis in oral-prostate axis exacerbates benign prostatic hyperplasia via IL-6/IL-6R pathway. Mil Med. Res. 11, 30. doi: 10.1186/s40779-024-00533-8, PMID: 38764065 PMC11103868

[B54] Wang S. Nie F. Yin Q. Tian H. Gong P. Ju J.. (2024). Periodontitis promotes tumor growth and immune evasion via PD-1/PD-L1. Cancer Immunol. Immunother. 74, 22. doi: 10.1007/s00262-024-03865-5, PMID: 39535607 PMC11561227

[B55] Wang H. Tang R. Luo S. Hou H. Liu J. Liu M.. (2025). Association of life’s crucial 9 score with benign prostatic hyperplasia: a cross-sectional study. J. Health Popul Nutr. 44, 163. doi: 10.1186/s41043-025-00925-z, PMID: 40390049 PMC12090484

[B56] Wei H. Tian G. Xu S. Du Y. Li M. Wang Y.. (2024). Evaluation of bi-directional causal association between periodontitis and benign prostatic hyperplasia: epidemiological studies and two-sample mendelian randomization analysis. Front. Genet. 15, 1326434. doi: 10.3389/fgene.2024.1326434, PMID: 38716069 PMC11075528

[B57] Wu L. Li B. H. Wang Y. Y. Wang C. Y. Zi H. Weng H.. (2019). Periodontal disease and risk of benign prostate hyperplasia: a cross-sectional study. Mil Med. Res. 6, 34. doi: 10.1186/s40779-019-0223-8, PMID: 31718713 PMC6852712

[B58] Yamashita K. Mizugishi K. Takaori-Kondo A. (2018). Familial mediterranean fever mutations in a patient with periodic episodes of systemic pain deriving from cancer bone metastases. Intern. Med. 57, 2901–2904. doi: 10.2169/internalmedicine.0431-17, PMID: 29780113 PMC6207823

[B59] Yang X. Chen H. Zhang J. Zhang S. Wu Y. S. Pang J. (2025). Association of cigarette use with risk of prostate cancer among US males: a cross-sectional study from NHANES 1999-2020. BMC Public Health 25, 608. doi: 10.1186/s12889-025-21863-9, PMID: 39948519 PMC11827229

[B60] Yang Y. Shigemura K. Maeda K. Moriwaki M. Chen K. C. Nakano Y.. (2024). The harmful effects of overlooking acute bacterial prostatitis. Int. J. Urol. 31, 459–463. doi: 10.1111/iju.15390, PMID: 38239011

[B61] Yang L. Wang L. Zheng Y. B. Liu Y. Bao E. H. Wang J. H.. (2025). Causal relationship between periodontitis and prostate diseases: a bidirectional Mendelian randomization study. Clin. Oral. Investig. 29, 127. doi: 10.1007/s00784-025-06211-w, PMID: 39945912

[B62] Yu Z. J. Yan H. L. Xu F. H. Chao H. C. Deng L. H. Xu X. D.. (2020). Efficacy and side effects of drugs commonly used for the treatment of lower urinary tract symptoms associated with benign prostatic hyperplasia. Front. Pharmacol. 11, 658. doi: 10.3389/fphar.2020.00658, PMID: 32457631 PMC7225336

[B63] Zattoni F. Ficarra V. Novara G. (2017). Risk stratification for benign prostatic hyperplasia. Urologia. 84, 153–157. doi: 10.5301/uro.5000220, PMID: 28315497

[B64] Zhang C. Fei Y. Li M. Li J. Tang M. Wang G.. (2025). Chitosan-P407-PNIPAM hydrogel loaded with AgNPs/lipid complex for antibacterial, inflammation regulation and alveolar bone regeneration in periodontitis treatment. Int. J. Biol. Macromol 307, 142080. doi: 10.1016/j.ijbiomac.2025.142080, PMID: 40107529

[B65] Zhao D. Z. Yang R. L. Wei H. X. Yang K. Yang Y. B. Wang N. X.. (2024). Advances in the research of immunomodulatory mechanism of mesenchymal stromal/stem cells on periodontal tissue regeneration. Front. Immunol. 15, 1449411. doi: 10.3389/fimmu.2024.1449411, PMID: 39830512 PMC11739081

